# High-quality wild barley genome assemblies and annotation with Nanopore long reads and Hi-C sequencing data

**DOI:** 10.1038/s41597-023-02434-2

**Published:** 2023-08-10

**Authors:** Rui Pan, Haifei Hu, Yuhui Xiao, Le Xu, Yanhao Xu, Kai Ouyang, Chengdao Li, Tianhua He, Wenying Zhang

**Affiliations:** 1 Research Center of Crop Stresses Resistance Technologies, Yangtze University, Jingzhou, 434025 China; 2 Western Crop Genetics Alliance, Western Australian State Agricultural Biotechnology Centre, College of Science, Health, Engineering and Education, Murdoch University, Murdoch, WA 6155 Australia; 3 Rice Research Institute, Guangdong Academy of Agricultural Sciences & Key Laboratory of Genetics and Breeding of High-Quality Rice in Southern China (Co-construction by Ministry and Province), Ministry of Agriculture and Rural Affairs & Guangdong Key Laboratory of New Technology in Rice Breeding & Guangdong Rice Engineering Laboratory, Guangzhou, 510640 China; 4 Grandomics Biotechnology Co., Ltd, Wuhan, 430076 China; 5 Hubei Collaborative Innovation Centre for Grain Industry, Yangtze University, Jingzhou, 434025 China; 6 Department of Primary Industries and Regional Development, South Perth, WA 6155 Australia; 7 MARA Key Laboratory of Sustainable Crop Production in the Middle Reaches of the Yangtze River (Co-construction by Ministry and Province), Yangtze University, Jingzhou, 434025 China

**Keywords:** Plant ecology, Plant evolution

## Abstract

Wild barley, from “Evolution Canyon (EC)” in Mount Carmel, Israel, are ideal models for cereal chromosome evolution studies. Here, the wild barley EC_S1 is from the south slope with higher daily temperatures and drought, while EC_N1 is from the north slope with a cooler climate and higher relative humidity, which results in a differentiated selection due to contrasting environments. We assembled a 5.03 Gb genome with contig N50 of 3.53 Mb for wild barley EC_S1 and a 5.05 Gb genome with contig N50 of 3.45 Mb for EC_N1 using 145 Gb and 160.0 Gb Illumina sequencing data, 295.6 Gb and 285.35 Gb Nanopore sequencing data and 555.1 Gb and 514.5 Gb Hi-C sequencing data, respectively. BUSCOs and CEGMA evaluation suggested highly complete assemblies. Using full-length transcriptome data, we predicted 39,179 and 38,373 high-confidence genes in EC_S1 and EC_N1, in which 93.6% and 95.2% were functionally annotated, respectively. We annotated repetitive elements and non-coding RNAs. These two wild barley genome assemblies will provide a rich gene pool for domesticated barley.

## Background & Summary

Barley (*Hordeum vulgare* L.), the fourth largest crop in terms of total cultivated area worldwide, is one of the earliest domesticated crops^1^. The cultivated barley is believed to be domesticated about 10,000 years ago from the wild progenitor *H. spontaneum*^2^. Beyond its importance as a major global crop, barley also serves as an invaluable model organism for research into crop domestication and adaptability due to its diploid status, relatively small genome within the Triticeae, and broad environmental adaptability^3,4^. A growing body of research highlights that during domestication, barley’s agronomic traits were selectively enhanced for efficient harvesting, maximized yield, and improved grain quality. In contrast, genetic variations crucial for survival under environmental stresses have been diminished or even eradicated^5^, posing significant challenges when breeding new resilient varieties in response to climate and environmental shifts., Wild barley (*Hordeum spontaneum* K. Koch), the ancestor of cultivated barley, has a wide eco-geographic distribution across highly diverse environments throughout Southwestern Asia^6^. The capacity of wild barley to withstand dry and hot conditions has significant implications for barley breeding, especially considering that a mere 40% of alleles present in wild barley are found within the gene pool of globally cultivated barley^7,8^. The wild barley population, therefore, can contribute a rich reservoir of genes tolerant to drought and heat, which can be introduced into domesticated barley - a feat made possible by the ease with which the two species can crossbreed. This paves the way for breeding cultivars resilient to climate change.

High-quality genome assembly is required for the exploitation of beneficial genetic variants in the wild barley^1,8^. With the advancements in sequencing technology, notable strides have been made in barley genomics. The draft sequence assembly of barley cultivar (cv.) Morex was reported in 2012, and it was further improved in 2017, especially in the centromeric region and highly repetitive region^7,9^, and again with significant improvement in continuity in 2021^10^. Besides, the draft genome and high-quality reference genome of Tibetan hulless barley have been publicly available in 2018, which significantly enriched barley genomic resources^8,11^. Recently, a barley pan-genome study reported the de novo assemblies for 20 representative barley worldwide accessions and revealed abundant structural variations among the genomes^12^, which underscore the need for high-quality wild barley assemblies in comparative genomic studies and future barley breeding initiatives.

The ‘Evolution Canyon’ model serves as an optimal micro-climatic divergence model between slopes, designed to understand the impact of climate and environmental changes on genomic adaptation and differentiation^13^. The sharp microclimatic divergence between the abutting slopes has been proposed to drive genomic adaptive divergence underpinnings of local adaptation, providing a unique system for comparative genomic study. High-quality genome assemblies of wild barley from micro-climatically contrasting sites can enrich the barley genome resources and provide genomic insight into the relationship between environmental selection and genome evolution. Here, we report two chromosome-scale assemblies for two wild barleys (EC_S1 from the south slope, EC_N1 from the north slope of Evolution Canyon in Mountains of Carmel, Israel), using the Oxford Nanopore long-read sequencing method, Hi-C chromosome conformation capture and Bionano-optical mapping technologies. With BUSCO, CEGMAG and GC-depth analysis, we demonstrate that the two assemblies are of high integrity and accuracy. Using the assemblies, we further predicted their genes, repetitive elements, and non-coding RNAs. The wild barley can provide a rich gene pool for stress-tolerant genes that might be introduced into domesticated barley, and our wild barley genomes will greatly facilitate such endeavours. The wild barley assemblies will also enable comparative genomic studies penetrating genomic evolution and adaptation of barley.

## Methods

### Sample preparation, library construction and sequencing

Seeds were collected from two samples, EC-S1 and EC-N1, at the South-facing slope and north-facing slope, respectively, of the “Evolution Canyon” in Mount Carmel, Israel, and were germinated and grown in the glasshouse at Yangtze University (Jingzhou, Hubei Province, China). Mature leaves were harvested for DNA extraction and sequencing. Genomic DNA was extracted following the CTAB method and purified with QIAGEN® Genomic kit (Cat#13343, QIAGEN, Germany). DNA quality and concentration were examined using a NanoDrop^TM^ 8000 spectrophotometer (Thermo Fisher Scientific, USA). DNA concentration was estimated with a Qubit® 4.0 Fluorometer (Thermo Fisher Scientific, USA).

For long-read sequencing, approximately 3–4 µg DNA per sample was used as input material for the ONT library preparations. After the sample was qualified, size-select of long DNA fragments was performed using the PippinHT system (Sage Science, USA). Next, the ends of DNA fragments were repaired, and A-ligation reactions were conducted with NEBNext Ultra II End Repair/dA-tailing Kit (Cat# E7546). The adapter in the SQK-LSK109 (Oxford Nanopore Technologies, UK) was used for further ligation reaction, and DNA library was measured by Qubit® 4.0 Fluorometer (Thermo Fisher Scientific, USA). About 700 ng DNA library was constructed and performed on a Nanopore PromethION sequencer instrument (Oxford Nanopore Technologies, UK) at the Genome Center of Grandomics (Wuhan, China).

A total of 295.6 Gb (~65× coverage of the estimated genome size) subreads in EC_S1 and 285.35 Gb (~65× coverage of the estimated genome size) subreads in EC_N1 were yielded for genome assembly. For the Illumina NovaSeq. 6000 platform, libraries for Illumina paired-end genome sequencing were constructed using Truseq Nano DNA HT Sample Preparation Kit (Illumina USA) following the standard manufacturer’s protocol (Illumina), and then sequenced with a paired-end sequencing strategy. Finally, we obtained 145.0 Gb (~32× coverage of the estimated genome size) in EC_S1 and 160.0 Gb (~36X coverage of the estimated genome size) clean data after quality inspection. For High-through chromosome conformation capture (Hi-C) sequencing, genomic DNA was extracted from the EC_S1 and EC_N1 sample. Thereafter, we constructed the Hi-C library and obtained sequencing data via the Illumina Novaseq/MGI-2000 platform to anchor hybrid scaffolds onto chromosome^14^. After quality control and filtration, 555.1 Gb (~122× coverage of the estimated genome size) clean data in EC_S1 and 514.5 Gb clean data in EC_N1 were obtained for the next analysis. Samples of roots and leaves (and young panicle) at the seedling, tillering and booting stage were used to collect transcriptome data by RNA sequencing for predicting the gene model.

Total RNA was extracted by grinding tissue in TRIzol reagent (TIANGEN, China) on dry ice and processed following the protocol provided by the manufacturer. The integrity of the RNA was determined with the Agilent 2100 Bioanalyzer (Agilent Technologies) and agarose gel electrophoresis. The purity and concentration of the RNA were determined with the Nanodrop^TM^ 8000 spectrophotometer (Thermo Fisher Scientific) and Qubit® 4.0 Fluorometer (Thermo Fisher Scientific, USA). cDNAs were prepared with DNA damage repair, end repair, and sequencing adapters ligation using SMRTbell Template Prep Kit 1.0 (Pacific Biosciences). The SMRTbell template was annealed to the sequencing primer, bound to polymerase, and sequenced on the PacBio Sequel platform using Sequel Binding Kit 3.0 (Pacific Biosciences) with 20 h movies. Finally, a total of 168.9 Gb clean data in EC_S1 and 111.8 Gb clean data in EC_N1 with filtration was yielded for further analysis.

For BioNano physical mapping, DNA extracted from EC_S1 and EC_N1 were subject to manufacturer-recommended protocols for library preparation (Plant DNA Isolation Kit,80003) and optical scanning provided by BioNano Genomics (https://bionanogenomics.com), with the labeling enzyme Direct Label Enzyme (DLE) (Bionano PrepDLS Labeling DNA Kit,80005). Labelled DNA samples were loaded and run on the Saphyr system (BioNano Genomics). Raw BioNano data were cleaned by removing molecules matching any of the following rules: length less than 150 kb, molecule signal-to-noise ratio less than 2.75, label signal-to-noise ratio less than 2.75, or label intensity greater 0.8. About 443.19 Gb and 311.01 Gb clean data were yielded after filtering with the parameter “Molecule length <150 kb” and “MinSites (/100 kb) <9”.

### *De novo* assembly of the wild barley genome

**Fig. 1:**
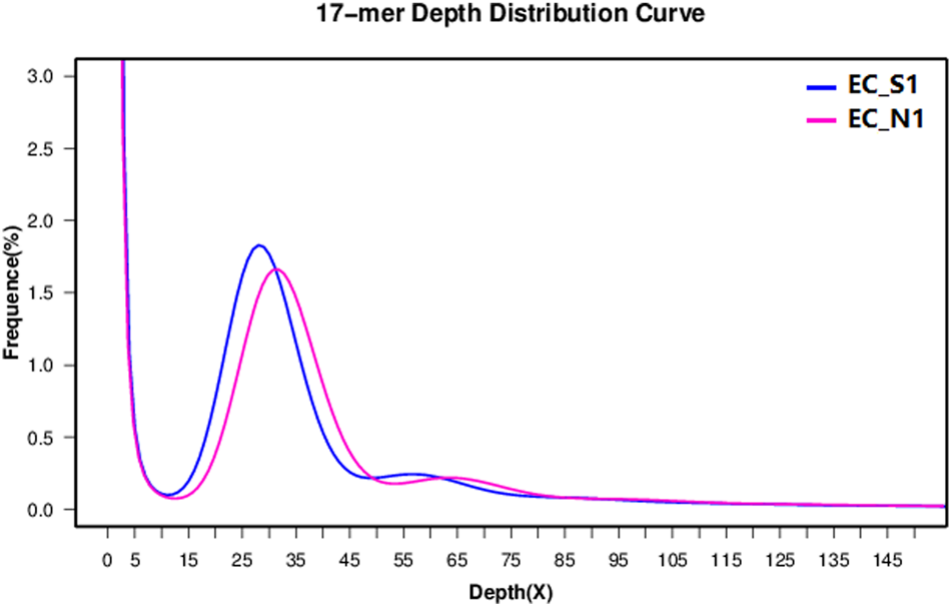
The *k*-mer distribution used to estimate the genome size of the wild barley EC_S1 and EC_N1. The distribution was determined based on the Jellyfish analysis using a *k*-mer size of 17.

To ensure reads are reliable, Illumina paired-ended sequenced raw reads for the genomic survey were first filtered using the Fastp v.0.20.0^15^ preprocessor (set to default parameters). To understand the genomic characteristics of EC_S1 and EC_N1, the *K*-mer analysis^16^ was performed using Illumina DNA data prior to genome assembly to estimate the genome size and heterozygosity. Briefly, quality-filtered reads were subjected to 17-mer frequency distribution analysis using the Jellyfish program^16^. The genome size was determined based on k-mer frequency distributions, using details from the peak depth and the count of 17-mers. Likewise, the heterozygosity rate was estimated utilizing the count of k-mers at half the peak depth and through simulation analysis using *A. thaliana* genome data as described in a previous publication^17^. The results indicated that the estimated genome sizes of EC_S1 and EC_N1 were 4.56 Gb and 4.40 Gb, respectively, both displaying low heterozygosity. (Fig. 1).

For de novo genome assembly, an ONT-only assembly was constructed by using an OLC (overlap layout-consensus)^18^/string graph method^19^ with NextDenovo. Considering the high error rate of ONT raw reads, the original subreads were first self-corrected using NextCorrect, thus obtaining 190.0 Gb (~38X coverage of the estimated genome size) and 172.8 Gb (~39× coverage of the estimated genome size) consistent sequences (CNS reads) in EC_S1 and EC_N1, respectively. Comparing CNS was then performed with the NextGraph module to capture correlations of CNS. Based on the correlation of CNS, 4.66 Gb preliminary genome with a contig N50 length of 3.26 Mb in EC_S1 and 4.66 Gb preliminary genome with a contig N50 length of 3.17 Mb in EC_N1 were obtained (Table 1). To improve the accuracy of the assembly, we refine the contigs with Racon^20^ using ONT long reads and Nextpolish using Illumina short reads with default parameters. Finally, we obtained a polish genome of 5.03 Gb with a contig N50 length of 3.53 Mb in EC_S1 and 5.05 Gb with a contig N50 length of 3.45 Mb in EC_N1 (Table 2).

**Table 1:** Statistics of EC_S1 and EC_N1 preliminary genome assembly.

Stat Type	EC_S1		EC_N1	
	Contig Length(bp)	Contig Number	Contig Length(bp)	Contig Number
N50	3,264,181	421	3,171,294	429
N60	2,617,547	580	2,530,446	593
N70	1,943,493	787	1,924,359	806
N80	1,285,538	1,079	1,275,833	1,102
N90	715,201	1,562	717,612	1,590
Longest	18,405,770	1	21,572,244	1
Total	4,656,798,638	2,593	4,659,696,944	2,628
Length > = 1 kb	4,656,798,638	2,593	4,659,696,944	2,628
Length > = 2 kb	4,656,798,638	2,593	4,659,696,944	2,628
Length > = 5 kb	4,656,798,638	2,593	4,659,696,944	2,628

**Table 2:** Statistics of the EC_S1 and EC_N1 polished genome assembly.

Parameter	EC_S1		EC_N1	
	Contig Length (bp)	Contig Number (#)	Contig Length (bp)	Contig Number (#)
N50	3,525,661	421	3,451,742	428
N60	2,827,275	579	2,752,099	592
N70	2,095,788	786	2,087,500	805
N80	1,389,397	1,078	1,385,479	1,100
N90	771,157	1,560	777,785	1,587
Longest	19,859,128	1	23,442,753	1
Total	5,025,137,494	2,593	5,052,015,165	2,628
Length > = 1 kb	5,025,137,494	2,593	5,052,015,165	2,628
Length > = 2 kb	5,025,137,494	2,593	5,052,015,165	2,628
Length > = 5 kb	5,025,137,494	2,593	5,052,015,165	2,628

The completeness of genome assembly was assessed using BUSCO v4.0.5 with single copy homologous genes in embryophyta_odb10 of OrthoDB database (Benchmarking Universal Single Copy Orthologs)^21^ and CEGMA v2 (Core Eukaryotic Gene Mapping Approach)^22^. 96.2% and 96.3% of complete BUSCOs were found in EC_S1 and EC_N1, respectively (Fig. S1). In addition, a total of 98.39% core genes in EC_S1 and 97.18% core genes in EC_N1 were detected among 248 core gene collections, suggesting high confidence in genome assembly in both EC_S1 and EC_N1 (Fig. S2). To evaluate the consistency of genome sequence, we aligned the second-generation sequencing data and the third-generation sequencing data to the polish genome by bwa v0.7.12-r1039^23^ and minimap2 vr41^24^. The results showed that the average depth of the second-generation sequencing data in the EC_ S1 and EC_ N1 was 28.22 and 31.22, respectively, and the coverage (depth > = 1×) was 85.97 and 84.88%. The average depth of the third-generation sequencing data in EC_ S1 and EC_ N1 was 55.12 and 43.83, respectively, and the coverage (depth > = 1×) was 99.80 and 99.62% (Table 3). GC-depth analysis showed that the GC content was distributed in 40%–50%, and the sequencing depth was concentrated in 40–80× in both EC_ S1 and EC_ N1 assemblies (Fig. S3). The corrected genome sequence was compared with NT library (Nucleotide Sequence Database, downloaded on 3^rd^ August 2018, https://ftp.ncbi.nlm.nih.gov/blast/db/FASTA/nt.gz) to determine the classification of the sequence, suggesting that there was a small amount of mitochondrial and chloroplast nucleic acids in the sequence but no exogenous pollution (Table 4).

**Table 3:** Genome sequence consistency and coverage.

Total Reads	Map Reads	Map Rate (%)	Average depth (X)	Coverage (Depth = 1X) (%)	Single nucleotide accuracy
971,244,971	969,319,898	99.8	28.22	85.97	99.997
1,071,625,423	1,069,602,506	99.81	31.22	84.88	99.996
18,481,260	18,474,324	99.96	55.12	99.8	—
11,802,366	11,798,906	99.97	43.83	99.62	—

**Table 4:** Genomic contamination assessment of EC_S1 and EC_N1 assemblies.

Assembly	Type	Contig Number	Contig Number ratio (%)	Contig Length (bp)	Contig Length ratio (%)
EC_S1	Viridiplantae	2,591	99.92	5,024,174,262	99.98
	Mitochondrion/Chloroplast	1	0.04	603,539	0.01
	Nohit	1	0.04	359,693	0.01
	Total	2,593	100	5,025,137,494	100
EC_N1	Viridiplantae	2,626	99.92	5,050,254,425	99.97
	Mitochondrion/Chloroplast	2	0.08	1,760,740	0.03
	Nohit	0	0	0	0
	Total	2,628	100	5,052,015,165	100

### Chromosome assembly by optical mapping and Hi-C data

**Fig. 2:**
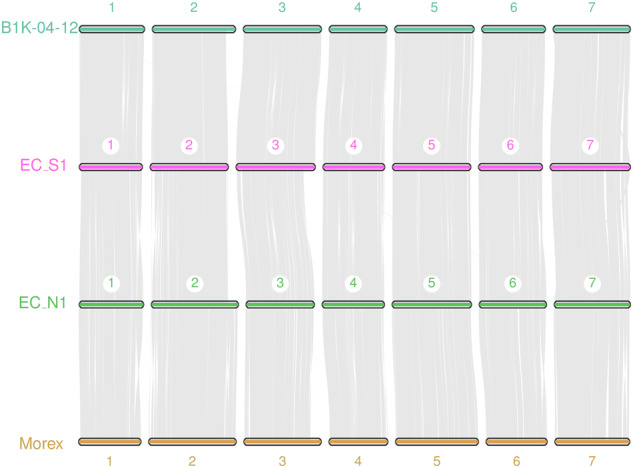
The collinearity analysis among assemblies of EC_S1, EC_N1, B1K-04–12 and Morex.

*De novo* assembly of BioNano molecules into genome maps was performed using the script pipelineCL.py in the BioNano Solve package v3.3 (BioNano Genomics). Hybrid scaffolds were assembled from ONT assembly and BioNano genome maps using the script hybridScaffold.pl in the Solve package. Finally, EC_S1’s genome super-scaffold size was 5.1 Gb with a scaffold N50 of 90.4 Mb and contig N50 of 1.67 Mb; EC_N1’s genome super-scaffold size was 5.2 Gb with scaffold N50 of 43.7 Mb and contig N50 of 1.59 Mb (Table 5). Compared to previously published barley genome assemblies, current genomes assemblies showed a great improvement in contig N50 and scaffold N50 (Table S1), and their quality was closed to the new version genome of Morex assembled by PacBio long-read (Table S2)^1,12^. For Hi-C auxiliary assembly, a total of 3.83 billion paired-end reads were generated from the libraries of EC_S1 and 3.54 billion from EC_N1. Then, quality controlling of Hi-C raw data was performed using HiC-Pro^25^ as in previous research. Firstly, low-quality sequences (quality scores < 20), adaptor sequences and sequences shorter than 30 bp were filtered out using Fastp^15^. The clean paired-end reads were then mapped to the draft assembled sequence using bowtie2 v2.3.2^26^ to get 759 million (39.63%) unique mapped paired-end reads in EC_S1 and 581 million (33.81%) in EC_N1. About 586 million (30.62%) valid interaction paired reads in EC_S1 and 436 million in EC_N1 were identified and retained by HiC-Pro^25^ from unique mapped paired-end reads for further analysis (Table 6). Invalid read pairs, including dangling-end, self-cycle, re-ligation, and dumped products, were filtered by HiC-Pro^25^. The 5.07 Gb scaffolds (98.58%) in EC_S1 and 5.10 Gb scaffolds (97.16%) in EC_N1 were further clustered, ordered, and oriented scaffolds onto the seven chromosomes by LACHESIS^27^, respectively (Table 7). According to the resulting Hi-C contact heatmap, mis-assemblies and mis-joins were manually corrected based on neighbouring interactions. The final assemblies were aligned to the previously reported barley genome assemblies of wild barley B1K-04–12 and cultivated barley Morex^12^ by Mummer v4.0^28^. Then the raw alignments results were further filtered by delta-filter from Mummer software^28^. The results were visualized by NGenomeSyn v2.0^29^, which demonstrated high collinearity across the majority of all chromosome regions. Furthermore, we identified abundant structural variations (SVs), including large fragment inversions (INVs) and insertions or deletions (INDELs) (refer to Fig. 2 and Fig. S4). These findings enrich the diversity of the barley genome resource.

**Table 5:** Statistics of scaffold constructed by BioNano in EC_S1 and EC_N1.

Samples	Stat Type	Scaffold Length (bp)	Scaffold Number (#)	Contig Length (bp)	Contig Number (#)	Gap Length (bp)	Gap Number (#)
EC_S1	N50	90,435,441	15	1,673,488	843	173,720	127
	N60	66,240,192	21	1,259,474	1,189	140,328	181
	N70	36,446,783	31	928,307	1,655	104,275	251
	N80	16,660,035	51	613,754	2,320	75,432	347
	N90	547,941	292	375,970	3,365	46,368	487
	Longest	275,161,632	1	9,363,529	1	1,469,128	1
	Total	5,110,963,655	3,671	5,025,137,494	6,981	85,826,161	3,310
	Length > = 1 kb	5,110,951,505	3,581	5,025,125,344	6,891	85,784,138	878
	Length > = 2 kb	5,110,917,100	3,558	5,025,090,939	6,868	85,771,526	870
	Length > = 5 kb	5,110,584,325	3,464	5,024,758,164	6,774	85,642,946	830
EC_N1	N50	43,706,098	29	1,588,206	905	970,159	36
	N60	24,896,804	45	1,191,192	1,272	407,276	64
	N70	14,063,939	74	850,038	1,773	226,986	119
	N80	5,480,950	134	576,932	2,499	142,755	212
	N90	518,178	509	352,017	3,618	75,821	372
	Longest	238,728,233	1	12,370,996	1	7,555,966	1
	Total	5,219,379,810	4,132	5,052,015,165	7,489	167,364,645	3,357
	Length > = 1 kb	5,219,364,283	4,036	5,051,999,638	7,393	167,326,492	897
	Length > = 2 kb	5,219,325,547	4,012	5,051,960,902	7,369	167,312,956	888
	Length > = 5 kb	5,218,959,372	3,908	5,051,594,727	7,265	167,164,119	847

**Table 6:** Valid paired end reads statistics of Hi-C data.

Sample	EC_S1	EC_N1
Unique Mapped Paired-end Reads	759,088,187	581,279,660
Dangling End Paired-end Reads	31,922,666	24,560,096
Self Circle Paired-end Reads	3,807,620	2,591,099
Dumped Paired-end Reads	132,876,819	113,956,204
Valid Paired-end Reads	586,484,335	436,245,999
Valid Rate (%)	77	75.05
Vailded reads of unique mapping(%)	31	25.38

**Table 7:** Chromosome length assembled by Hi-C data in EC_S1 and EC_N1.

Chromosome	EC_S1		EC_N1	
	Size (bp)	Scaffold Number (#)	Size (bp)	Scaffold Number (#)
LG01 (Chr2H)	701,205,537	58	718,190,815	83
LG02 (Chr7H)	679,094,545	57	654,994,973	86
LG03 (Chr3H)	675,898,809	47	610,452,210	114
LG04 (Chr4H)	668,531,449	95	584,827,964	89
LG05 (Chr5H)	632,171,286	50	572,391,443	39
LG06 (Chr6H)	612,997,366	54	560,827,502	91
LG07 (Chr1H)	554,163,281	23	349,920,086	22
Total	4,524,062,273	384	4,051,604,993	524

### Gene model prediction and functional annotation

We first annotated the tandem repeats using the software GMATA^30^ and Tandem Repeats Finder (TRF)^31^, where GMATA identifies the simple repeats sequences (SSRs) and TRF recognizes all tandem repeat elements in the whole genome. Transposable elements (TE) in the EC_S1 and EC_N1 genomes were then identified using a combination of ab initio and homology-based methods. For further identification of the repeats throughout the genome, RepeatMasker v2.0.1^32^ was applied to search for known and novel TEs by mapping sequences against the de novo repeat library and Repbase TE library (version 20180826)^33^. Overlapping transposable elements belonging to the same repeat class were collated and combined. The repeat elements were annotated and shown in Table 8.

**Table 8:** Characterization of wild barley TE annotation in wild barley EC_S1 and EC_N1.

Class	Order	Super family	EC_S1			EC_N1		
			Number of elements	Length of sequence (bp)	Percentage of sequence (%)	Number of elements	Length of sequence (bp)	Percentage of sequence (%)
Class I	*tatal*		4,978,332	4,068,129,400	79.6	4,723,019	4,070,454,371	77.99
	LTR	*total*	4,687,283	3,961,835,992	77.52	4,451,964	3,967,760,212	76.02
		Unknown	2,028,714	1,258,880,928	24.63	1,935,231	1,222,171,928	23.42
		Copia	719,996	837,900,974	16.39	650,395	794,223,165	15.22
		Gypsy	1,930,780	1,853,447,741	36.26	1,855,837	1,946,140,147	37.29
		Ngaro	5,420	10,505,226	0.21			
		Other	2,373	1,101,123	0.02	10,501	5,224,972	0.1
	LINE	*total*	226,254	99,136,722	1.94	242,326	99,764,545	1.91
		Unknown	144,737	36,996,261	0.72	156,002	39,317,430	0.75
		L1	77,807	60,197,197	1.18	82,685	58,346,236	1.12
		Other	3,710	1,943,264	0.04	3,639	2,100,879	0.04
	SINE	*total*	64,795	7,156,686	0.14	28,729	2,929,614	0.06
		Unknown	64,665	7,150,037	0.14			
		Other	130	6,649	0	28,729	2,929,614	0.06
Class II	*total*		994,800	390,247,909	7.64	1,067,975	416,959,633	7.99
	DNA	*total*	869,197	365,992,210	7.16	930,184	389,472,730	7.46
		Unknown	413,075	97,864,858	1.91	486,434	100,295,321	1.92
		CMC-EnSpm	332,344	230,347,500	4.51	318,045	249,528,462	4.78
		MULE-MuDR	36,509	16,457,049	0.32	36,575	17,892,859	0.34
		PIF-Harbinger	35,961	12,689,209	0.25	34,867	12,358,068	0.24
		Other	51,308	8,633,594	0.17	54,263	9,398,020	0.18
	RC	Other	26,312	5,377,294	0.11	15,817	2,125,122	0.04
Total TEs			5,973,132	4,458,377,309	87.23	5,790,994	4,487,414,004	85.98
Tandem Repeats	*total*		169,508	11,650,363	0.23	170,835	11,744,363	0.23
	SSR		68,608	826,702	0.02	71,838	864,442	0.02
	tandem_repeat		100,900	10,823,661	0.21	98,997	10,879,921	0.21
Unknown			322,655	68,422,508	1.34	307,877	56,497,367	1.08
Simple repeats			17,136	4,940,437	0.1	17,083	4,370,082	0.08
Other			54,300	11,252,823	0.22	11,779	1,365,226	0.03
Low complexity			3,462	634,097	0.01	4,904	667,464	0.01
Total Repeats			6,540,193	4,555,277,537	89.13	6,303,472	4,562,058,506	87.41

Three independent approaches, including ab initio prediction, homology search, and reference guided transcriptome assembly, were used for gene prediction in a repeat-masked genome^34^. In detail, GeMoMa v1.3.1^35^ was used to align the homologous protein sequences from related species to the assembly and then got the gene structure information, which was homolog prediction. For RNA-seq based gene prediction, filtered mRNA-seq reads were aligned to the reference genome using STAR (default)^36^. The transcripts were then assembled using Stringtie v2.1.4^37^ and open reading frames (ORFs) were predicted using Program to Assemble Spliced Alignments (PASA)^38^. For the de novo prediction, RNA-seq reads were de novo assembled using Stringtie and analyzed with PASA to produce a training set. Augustus v2.5.5^39^ with default parameters was then utilized for ab initio gene prediction with the training set. Finally, EVidenceModeler (EVM)^40^ was used to produce an integrated gene set of which genes with TE were removed using Transposon PSI package^41^ and the miscoded genes were further filtered. According to Mascher *et al*.^7^, high-confidence (HC) gene was defined as genes that had a significant BLAST hit to reference proteins and representative proteins had a similarity to the respective template sequence above a threshold which was determined on the basis of the origin of template sequences (>60% for *Arabidopsis thaliana*, sorghum and rice, >65% for *Brachypodium distachyon*, and >85% for barley). Finally, a total of 39,179 high-confidence and 20,936 low-confidence protein-coding genes were identified in EC_S1 genome, and 38,373 high-confidence and 20,243 low-confidence protein-coding genes in EC_N1 (Table 9).

**Table 9:** The summary of gene annotation in the EC_S1 and EC_N1 assemblies.

Annotation	Methods	EC_S1		EC_N1	
		Number (#)	Percentage (%)	Number (#)	Percentage (%)
Structure annotation	*De novo*	67,693	112.61	64,389	109.85
	Homology	47,152	78.44	47604	81.21
	RNA-seq	21,019	34.96	20026	34.16
	High-confidence	39,179	65.17	38373	65.47
	Low-confidence	20936	34.83	20243	34.53
	Total	60,115	100	58616	100
Functional annotation	KOG	23,722	39.46	23,687	40.41
	KEGG	16,549	27.53	16,673	28.44
	NR	55,919	93.02	55,417	94.54
	SwissProt	35,219	58.59	35,574	60.69
	GO	26,264	43.69	26,512	45.23
	Overall_annotated	56,261	93.59	55,772	95.15

Gene functional information, motifs and domains of their proteins were assigned by comparing with public databases including SwissProt^42^, NCBI non-redundant protein sequences (nr)^43^, Kyoto Encyclopedia of Genes and Genomes (KEGG)^44^, Clusters of orthologous groups for eukaryotic complete genomes (KOG)^45^ and Gene Ontology (GO)^46^. The putative domains and GO terms of genes were identified using the InterProScan program^47^ with default parameters. For the other four databases, BLASTp^48^ was used to compare the EvidenceModeler-integrated protein sequences against the four well-known public protein databases with an E-value cutoff of 1e-05 and the results with the hit with the lowest E value were retained. Results from the five database searches were concatenated, leading to a total of 56,261 (93.59%) genes in EC_S1 and 55,772 (95.15) genes in EC_N1 with function annotation (Table 9).

### Annotation of non-coding RNA genes

To obtain the ncRNA (non-coding RNA), we used two strategies: searching against a database and predicting with a model. Transfer RNAs (tRNAs) were predicted using tRNAscan-SE v2.0.6^49^ with eukaryote parameters. MicroRNA, rRNA, small nuclear RNA, and small nucleolar RNA were detected using Infernal cmscan^50^ to search the Rfam database^51^. The rRNAs and their subunits were predicted using RNAmmer^52^. Finally, a total of 1,163 and 888 rRNA was identified in EC_S1 and EC_N1, respectively. Moreover, total of 7770 ncRNA was identified in EC_S1, including 1180 snRNA (0.0024%), 6188 miRNA (0.0158%), 229 spliceosomal (0.0007%) and 173 other (0.0005%); 7701 ncRNA was identified in EC_N1, including 1065 snRNA (0.0021%), 6246 miRNA (0.0156%), 225 spliceosomal (0.0007%) and 165 other (0.0005%). In addition, 1913 and 2039 tRNA were detected in EC_S1 and EC_N1, covering all 20 anti-codons types of amino acids (Table 10).

**Table 10:** Summary of non-coding RNA in the EC_S1 and EC_N1 assemblies.

Type		EC_S1				EC_N1			
		Number (#)	Average length (bp)	Total length (bp)	Percentage (%)	Number (#)	Average length (bp)	Total length (bp)	Percentage (%)
rRNA	Total	1,163	306.35	356,281	0.007	888	6302.03	368966	0.0071
	18 S	43	1,819.65	78,245	0.0015	50	1772.52	88626	0.0017
	28 S	34	4,467.53	151,896	0.003	44	4260.2	187449	0.0036
	5 S	1,080	115.96	125,240	0.0025	790	116.81	610	0
	5.8 S	6	150	900	0	4	152.5	92281	0.0018
ncRNA	Total	7,770	127.53	990,911	0.0194	7701	544.67	984269	0.0189
	other	173	146.58	25,358	0.0005	165	154.86	25552	0.0005
	snRNA	1,180	105.15	124,074	0.0024	1065	105.16	111993	0.0021
	miRNA	6,188	130.12	805,169	0.0158	6246	129.99	811926	0.0156
	spliceosomal	229	158.56	36,310	0.0007	225	154.66	34798	0.0007
regulatory	cis-regulatory	171	45.96	7,859	0.0002	205	49.34	10115	0.0002
tRNA	tRNA	1,913	75.2	143,855	0.0028	2039	75.34	153619	0.0029

## Data Records

The EC_S1 and EC_N1 genome sequence are available at NCBI database under Bioproject accession PRJNA947680^53,54^. RNA-seq (The samples’ information are showed in Table S3), NGS, Hi-C, and Nanopore data sets are available at NCBI under Bioproject accession PRJNA748178^55^. Bionano data sets are available at NCBI Supplementary Files under accession SUPPF_0000004010 (EC_S1) and SUPPF_0000004011 (EC_N1)^55^. The genome annotation GFF3, CDS sequences, and protein sequences are available at figshare^56^.

## Technical Validation

### DNA and RNA integrity

The quality of DNA and RNA molecules and libraries was examined before genome and transcriptome sequencing. The DNA degradation and contamination of the extracted DNA were monitored on 1% agarose gels. DNA purity was then inspected using NanoDrop™ 8000 spectrophotometer (Thermo Fisher Scientific, USA), of which OD260/280 ranged from 1.8 to 2.0 and OD 260/230 was between 2.0 to 2.2. Finally, DNA concentration was further measured by Qubit® 4.0 Fluorometer (Thermo Fisher Scientific, USA). The integrity of the RNA was determined with the Agilent 2100 Bioanalyzer (Agilent Technologies) and agarose gel electrophoresis. The purity and concentration of the RNA were determined with the Nanodrop^TM^ 8000 spectrophotometer (Thermo Fisher Scientific, USA) and Qubit® 4.0 Fluorometer (Thermo Fisher Scientific, USA). Only the high-quality RNA sample (OD260/280 = 1.8~2.2, OD260/230 ≥ 2.0, RIN ≥ 7, >1 μg) was used to construct the sequencing library.

### Assessment of the genome assembly

After using BUSCO and CEGMA to evaluate genome integrity, we have also evaluated the accuracy of the genome. All the Illumina paired-end reads were mapped to the assembled genome using bwa 0.7.12-r1039 (default)^22^, and the mapping rate, as well as genome coverage of sequencing reads were assessed. Then samtools v1.4^57^ and bcftools v2.29.2^58^ were used to calculate the homozygous and heterozygous mutation sites corresponding to the samples. Homozygous sites were regarded as genomic error sites to calculate the single base error rate. The accuracy of genomic single base was 99.997% (depth > = 5x) in EC_S1 and 99.996% (depth > = 5x) in EC_N1. The Minimap2 r41 (-x map-ont)^23^ was used to map all long-reads back to the genome, to calculate mapping rate, coverage, and GC content. the draft genome assemblies were submitted to the NT library (Nucleotide Sequence Database, downloaded on 3^rd^ August, 2018, https://ftp.ncbi.nlm.nih.gov/blast/db/FASTA/nt.gz) and aligned sequences were eliminated to remove the mitochondria sequences in the assemblies. The results showed that most of the sequences were aligned with the target species, indicating that there was no external contamination in the assembled genome.

**Fig. 3:**
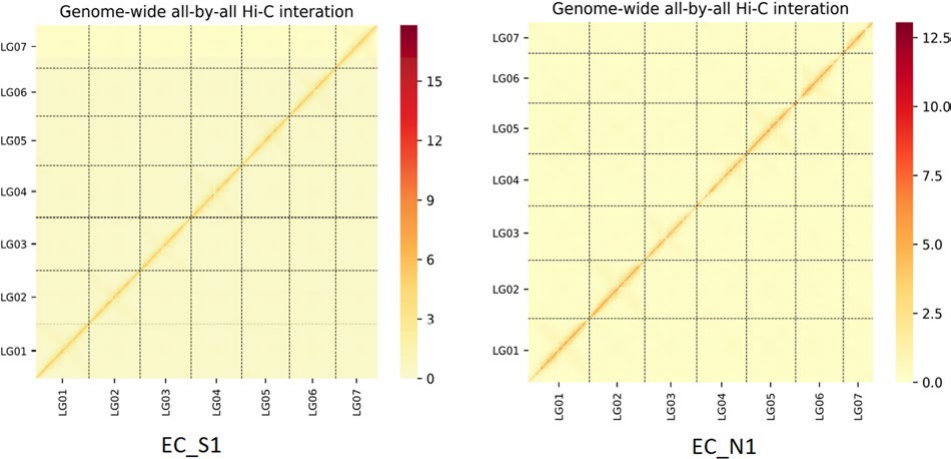
Heat map of chromosomes interactions by Hi-C sequence of wild barley EC_S1 and EC_N1. LG1-LG7 represent Chr2H, Chr7H, Chr3H, Chr4H, Chr5H, Chr6H, Chr1H, respectively. The horizontal and vertical coordinates represent the order of each ‘bin’ on the corresponding chromosome.

Finally, the seven chromosomes of EC_S1 and EC_N1 assemblies were evaluated. The genome with chromosomes aligned by Hi-C data was divided into ‘bin’ (in a length of 100 KB). The number of Hi-C read pairs covered by any two ‘bins’ was used to define the signal for the interaction between those ‘bins’^27^, and the heat map of Hi-C interaction of chromosomes was made by HiCPlotter.py script in Python v2.7 (Fig. 3). This figure shows that the intensity of interaction in the diagonal position was higher than that in the non-diagonal position, and there was no obvious noise outside the diagonal, indicating that the chromosomes assembly of both EC_S1 and EC_N1 were high-quality.

### Supplementary information

Figure S1

Figure S2

Figure S3

Figure S4

Supplementary Tables S3

Supplementary Tables S1

Supplementary Tables S2

Supplementary Figure

## Data Availability

No specific code or script was used in this work. All commands used in the processing were executed according to the manual and protocols of the corresponding bioinformatics software.
