# Supplementary figures and images for: High-quality wild barley genome assemblies and annotation with Nanopore long reads and Hi-C sequencing data
Source: Sci Data. 2023 Aug 10;10:535. doi: 10.1038/s41597-023-02434-2 (PMC10415357; doi:10.1038/s41597-023-02434-2)

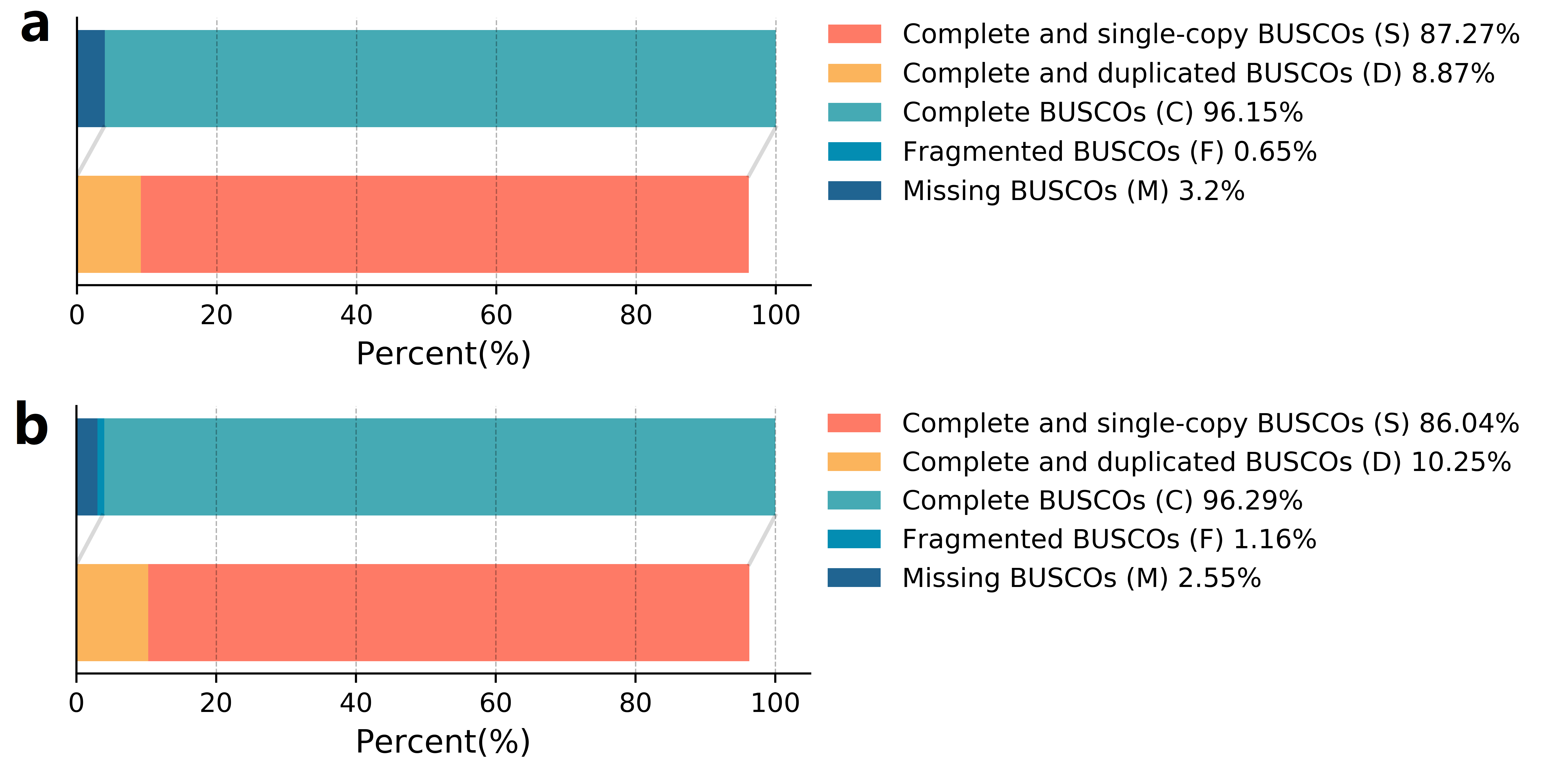

Supplement: Supplementary file 1 — Figure S1 [file 41597_2023_2434_MOESM1_ESM.png]

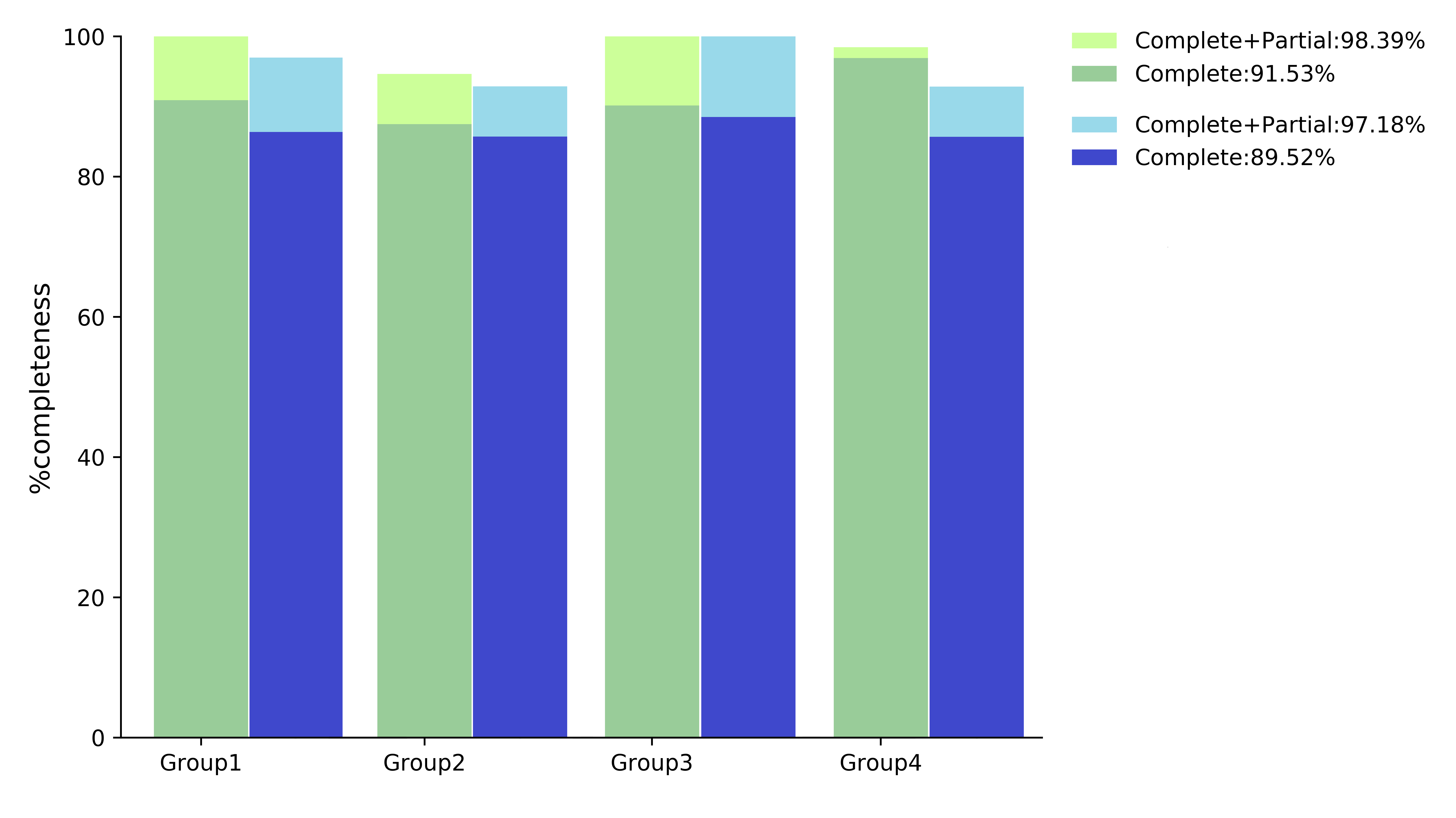

Supplement: Supplementary file 2 — Figure S2 [file 41597_2023_2434_MOESM2_ESM.png]

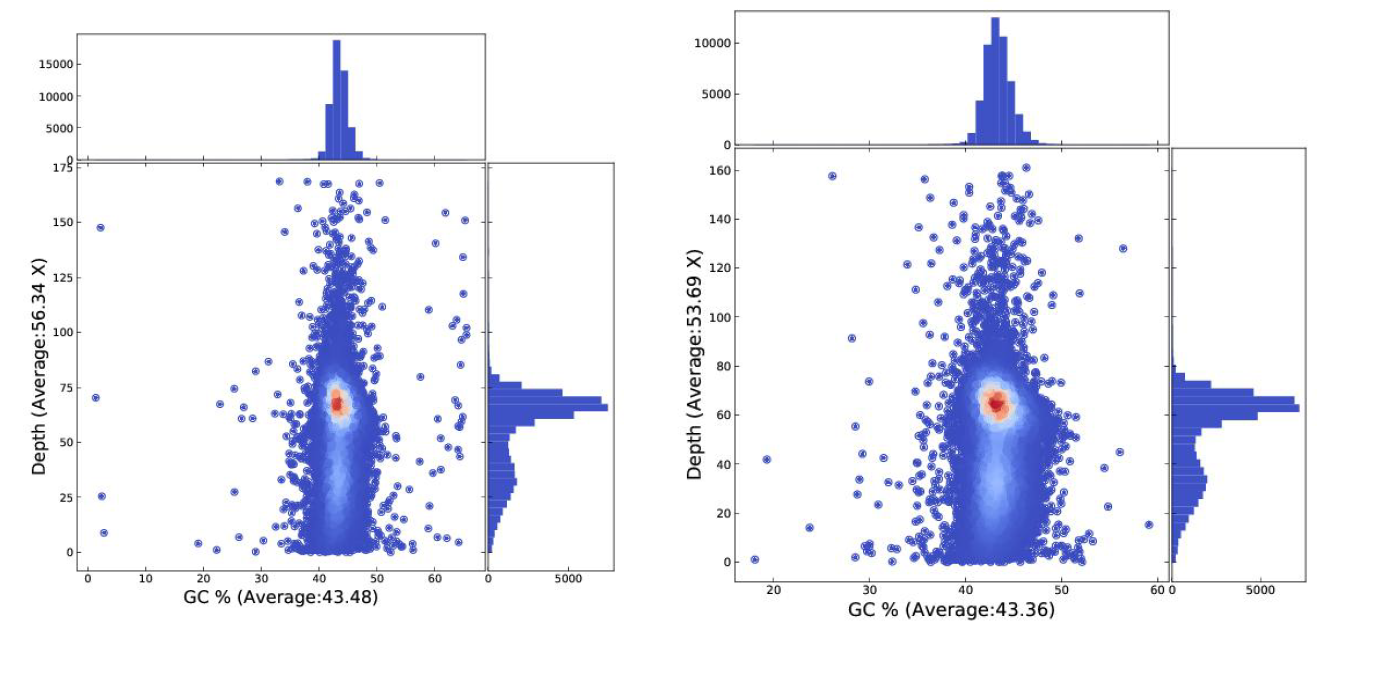

Supplement: Supplementary file 3 — Figure S3 [file 41597_2023_2434_MOESM3_ESM.png]

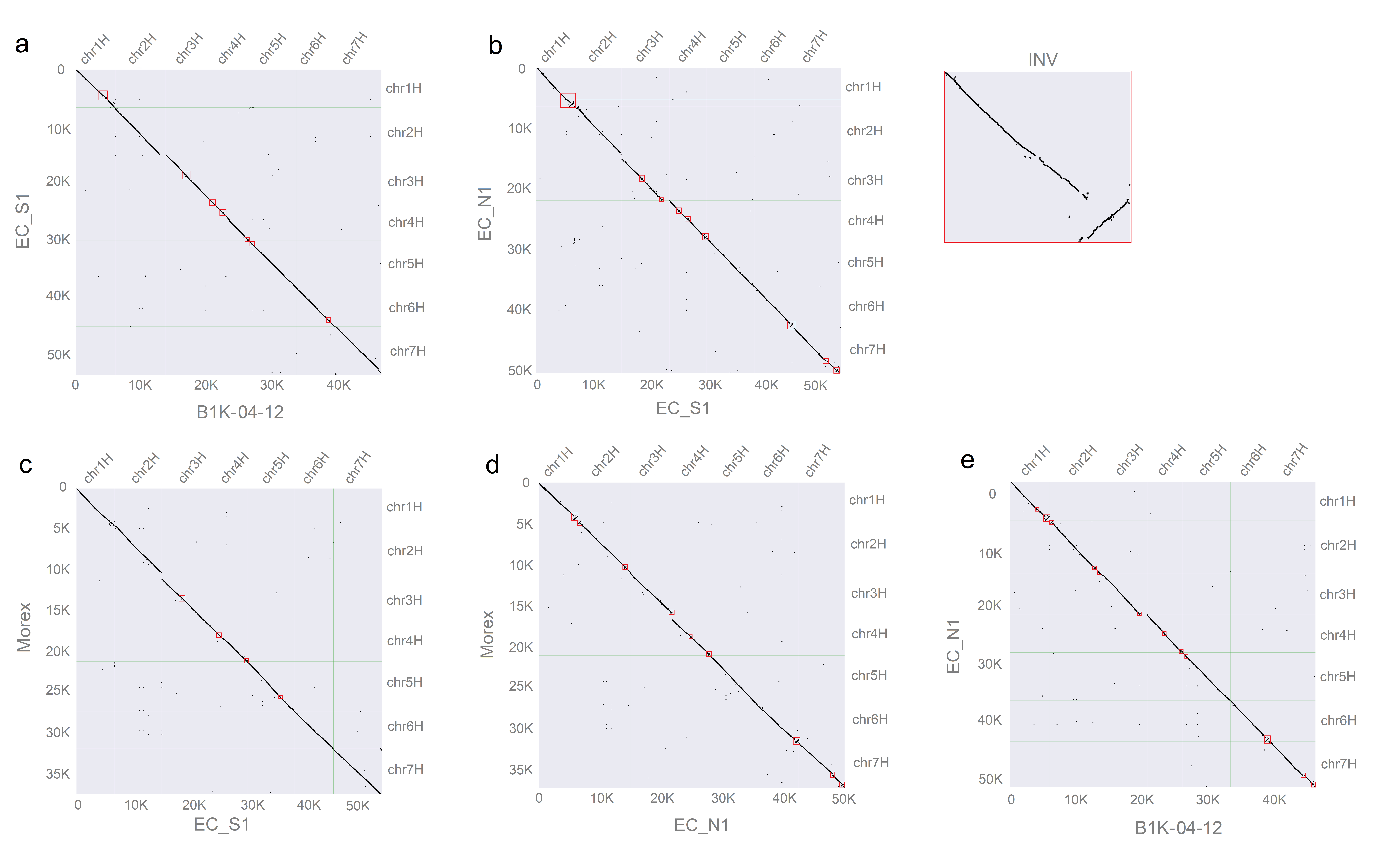

Supplement: Supplementary file 4 — Figure S4 [file 41597_2023_2434_MOESM4_ESM.png]
