# Supplementary material for: High-quality wild barley genome assemblies and annotation with Nanopore long reads and Hi-C sequencing data
Source: Sci Data. 2023 Aug 10;10:535. doi: 10.1038/s41597-023-02434-2 (PMC10415357; doi:10.1038/s41597-023-02434-2)
Supplement: Supplementary file 8 — Supplementary Figure [file 41597_2023_2434_MOESM8_ESM.docx]

**Figure legends**

**Fig. S1** BUSCO evaluation statistics of assemblies of the wild barley EC_S1 (a) and EC_N1 (b).

**Fig. S2** CEGMA evaluation statistics of wild barley genome assemblies.

**Fig. S3** GC-depth distribution of EC_S1 and EC_N1. The X-axis is GC content, and the Y-axis is depth. These two values are counted per 100 kb of genome.

**Fig. S4** The comparisons among assemblies of EC_S1, EC_N1, B1K-04-12 and Morex. (a) EC_S1 and B1K-04-12; (b) EC_S1 and EC_N1, the red box showed an example of large segment inversion (INV); (c) EC_S1 and Morex; (d) EC_N1 and Morex; (e) EC_N1 and B1K-04-12.
